# Establishment and validation of a method for determining anti-Xa factor potency of enoxaparin sodium

**DOI:** 10.7717/peerj.19437

**Published:** 2025-05-06

**Authors:** En Zhang, Hanyan Zou, Yandong Dong, Bing Liu, Xiaorong Yang

**Affiliations:** 1 Chongqing Institute for Food and Drug Control, Chongqing, China; 2 Key Laboratory for Innovative Biological Products Development and Evaluation, Chongqing, China; 3 College of Pharmaceutical Sciences Southwest University, Chongqing, China

**Keywords:** Enoxaparin sodium, Anti-Xa factor, Potency determination, Method validation, Quality control

## Abstract

This study successfully established and validated an efficient, reliable, and user-friendly method for determining the anti-Xa factor potency of enoxaparin sodium. Comprehensive validation experiments demonstrated the method’s excellent performance in terms of specificity, linearity, precision, accuracy, and robustness. The method exhibited a linear detection range of 0.054–0.192 IU/mL with a strong correlation coefficient, and its precision, robustness, and consistency with the European Pharmacopoeia method were all within 2.0% relative standard deviation (RSD). These results indicate high reproducibility and strong applicability, making the method suitable for seamless transfer between laboratories. Accuracy experiments revealed recovery rates ranging from 98.0% to 102.0%, confirming the reliability of the results. The validation design and performance of this method comply with the requirements of ICH guidelines and the Chinese Pharmacopoeia. Compared with traditional methods, this approach significantly reduces sample and reagent consumption, lowers experimental costs, and optimizes operational procedures, offering a low-cost, high-efficiency tool for quality control. These findings provide essential technical support for the production and quality monitoring of enoxaparin sodium and serve as valuable references for the development and validation of quality standards for similar biological products.

## Introduction

Enoxaparin sodium, a low molecular weight heparin (LMWH), is typically derived from the benzyl ester derivative of heparin extracted from porcine intestinal mucosa through alkaline depolymerization, with a molecular weight range of approximately 3,800–5,000 Daltons (Council of Europe, 2024a, 2024c). Anti-Xa factor activity is a critical quality attribute of enoxaparin sodium’s biological activity, serving as a key parameter for batch release testing and stability assessments by manufacturers, as well as a primary focus for regulatory authorities (International Conference on Harmonisation of Technical Requirements for Registration of Pharmaceuticals for Human Use, 1999, 2008, 2023b). Consequently, establishing a reliable detection method with high stability and consistency is essential to ensure the quality of enoxaparin sodium.

Currently, anti-Xa factor potency assays are primarily based on the substrate chromogenic detection method specified in the European Pharmacopoeia (Council of Europe, 2024c) or methods independently developed by individual manufacturers. The anti-Xa activity assay of enoxaparin sodium is based on its specific binding to antithrombin III (AT-III), which significantly enhances the inhibitory effect of AT-III on factor Xa. In this reaction system, residual uninhibited factor Xa hydrolyzes the chromogenic substrate S-2765 (N-α-benzyloxycarbonyl-D-arginyl-L-glycyl-L-arginine-p-nitroaniline dihydrochloride), releasing p-nitroaniline, which exhibits characteristic absorption at 405 nm. By measuring changes in absorbance at this wavelength, the degree of factor Xa inhibition can be indirectly assessed, thereby enabling the calculation of enoxaparin sodium’s anti-Xa activity. Specifically, this method quantifies anti-Xa activity in terms of relative potency by comparing the dose-response curves of the reference standard and test samples. Despite their widespread use, these methods often suffer from inconsistencies in results due to variations in equipment, high operator-dependent procedural demands, laboratory staff turnover, and differences in reagent sources (Ignjatovic et al., 2007; Kitchen, Theaker & Preston, 2000; Kovacs et al., 1999). Such inconsistencies compromise the effectiveness of regulatory oversight and weaken the reliability of drug quality control. Addressing these challenges requires the development of a robust, simplified, and highly reproducible anti-Xa factor potency assay, which is critical for ensuring drug quality and improving inter-laboratory comparability and consistency.

This study aims to integrate multiple manufacturer-developed methods to establish a calibration-based anti-Xa potency assay using internationally recognized reference standards. The proposed method is designed to be simple, operable by a single technician without specialized equipment, and capable of enhancing consistency across different laboratories. To ensure the reliability and feasibility of this method, the study adheres to the requirements of ICH Q2 (International Conference on Harmonisation of Technical Requirements for Registration of Pharmaceuticals for Human Use, 2023a) and the 2020 edition of the Chinese Pharmacopoeia*’s Analytical Method Validation Guideline* (9101) (Chinese Pharmacopoeia Commission, 2020). Comprehensive validation will include assessments of the method’s stability, precision, and accuracy. The ultimate goal of this study is to develop a practical and standardized detection protocol for evaluating the production quality of enoxaparin sodium, providing manufacturers and regulatory agencies with a robust, consistent, and scientifically validated quality control tool.

## Methods

### Reagents and materials

The low molecular weight heparin biological reference standard (Batch No. 11) provided by the European Directorate for the Quality of Medicines (EDQM) was used in this study. This reference standard has an anti-factor Xa activity of 110 IU/mL and is specifically designed for anti-factor Xa activity assays. Detailed information can be found in the monograph on low molecular weight heparins in the European Pharmacopoeia (*Ph. Eur*.).

The instruments used in this study included a Thermo Varioskan Flash UV-Vis spectrophotometer for reading enzyme-linked immunosorbent assay (ELISA) results and a Shimadzu UV-2600 UV-Vis spectrophotometer for measuring the absorbance of mixed solutions using semi-micro cuvettes.

### Reagent preparation

***Antithrombin III solution*:** Provided by BioWill (Batch No. 230823), containing 10 IU per vial. The solution was diluted to a final concentration of 0.5 IU/mL using a Tris (hydroxymethyl) aminomethane sodium chloride buffer (pH 7.4) (Council of Europe, 2024d).

***Bovine factor Xa solution*:** Supplied by BioWill (Batch No. 220810), with a concentration of 2.5 IU per vial. It was diluted to 0.23 IU/mL using a pH 7.4 buffer solution (Council of Europe, 2024b).

***Chromogenic substrate solution (S-2765)*:** Provided by BioWill (Batch No. 230907), containing 10 mg per vial. Initially diluted to a concentration of 3 mM with purified water, it was further diluted to 0.5 mM with a pH 8.4 buffer solution before use.

***30% Acetic acid solution*:** Prepared by diluting 30 mL of glacial acetic acid with ultrapure water (R) to a total volume of 100 mL.

### Enoxaparin sodium samples

The enoxaparin sodium samples were provided by Yino Pharma Limited, with batch numbers YN001, YN002, and YN003. Initial experiments estimated their anti-factor Xa activity at 100 IU/mg, which was used for solution dilution calculations (see Table 1). Batch YN001 was used for method validation. Upon confirming the feasibility of the method, further tests were conducted on batches YN001, YN002, and YN003 to evaluate the method’s applicability and reproducibility.

**Table 1:** Preparation of series test solutions for anti-Xa factor potency assay of enoxaparin sodium. This table outlines the detailed preparation scheme for the series of test solutions used in the anti-Xa factor potency assay. The solutions were prepared by diluting the initial enoxaparin sodium samples to achieve the desired concentrations for validation experiments. The table includes information on sample weights, estimated potency, dilution volumes, and final concentrations. Since the test sample is in solid form, the initial concentration of the solution may vary slightly due to differences in the precise weighing during sample preparation. Consequently, the volume of the initial solution required to prepare the 11 IU/mL TC_0_ solution may fluctuate, as indicated by the variable volume of 0.124 mL marked with an asterisk (*) in the table.

ID	Estimated activity (IU/mL)	Be diluted (mL)			Diluents (mL)	
Test sample T	100.25	/				
TC_0_	11	Test Sample T	0.124*	+	Water *R*	1.0
TC_1_	1.0	TC_0_ Solution	0.1	+	Water *R*	1.0
T_1_	0.16	TC_2_ Solution	0.16	+	pH7.4	0.84
T_2_	0.12	T_1_ Solution	0.75	+	pH7.4	0.25
T_3_	0.09	T_2_ Solution	0.75	+	pH7.4	0.25
T_4_	0.0675	T_3_ Solution	0.75	+	pH7.4	0.25

**Note:**

Abbreviations: pH7.4, Tris (hydroxymethyl)aminomethane sodium chloride buffer solution pH7.4. Water *R*, Reagent Water, which is a high-purity water that complies with pharmacopoeial standards and is suitable for use in experiments and assays, as opposed to ordinary water.

### Solution preparation

The dilution schemes for test and standard solutions were prepared according to Tables 1 and 2.

**Table 2:** Preparation of series standard solutions for anti-Xa factor potency assay of enoxaparin sodium. This table provides the detailed preparation scheme for the series of standard solutions used in the anti-Xa factor potency assay. The standard solutions were prepared by diluting the low molecular weight heparin biological reference standard (Batch No. 11, EDQM) to achieve the desired concentrations for calibration and validation experiments. The table includes information on the reference standard potency, dilution volumes, and final concentrations.

ID	Labeled activity (IU/mL)	Be diluted (mL)			Diluents (mL)	
Standard S	110	1.0 ml of water *R* for reconstitution				
SC_0_	11	Standard S	1.0	+	Water *R*	9.0
SC_1_	1.0	SC_0_ Solution	0.1	+	Water *R*	1.0
S_1_	0.16	SC_2_ Solution	0.16	+	pH7.4	0.84
S_2_	0.12	S_1_ Solution	0.75	+	pH7.4	0.25
S_3_	0.09	S_2_ Solution	0.75	+	pH7.4	0.25
S_4_	0.0675	S_3_ Solution	0.75	+	pH7.4	0.25
SC_Max_	0.192	SC_2_ Solution	0.192	+	pH7.4	0.808
SC_Min_	0.054	SC_2_ Solution	0.054	+	pH7.4	0.946

For experiments validating specificity, linearity, and range, eight solutions were prepared: six standard solutions of different concentrations were prepared, including SC_Max_, SC_Min_, S1, S2, S3, and S4, an enoxaparin sodium test solution T4 and a blank control. The blank control consisted of pH 7.4 buffer without enoxaparin sodium to assess matrix interference effects.

Other validation experiments, including precision, accuracy, and repeatability assessments, utilized pH 7.4 buffer, four concentrations gradients of enoxaparin sodium solutions (T1, T2, T3, and T4), and four concentrations gradients of standard solutions (S1, S2, S3, and S4). In all experiments, pH 7.4 buffer served as the negative control. A dual-well parallel design was employed, with the negative control wells positioned at both ends of the sample arrangement sequence to minimize experimental errors.

Enoxaparin sodium, as a solid sample, had its solution concentrations calculated based on the initial sample weight. Consequently, the concentration of each batch varied. The estimated concentration of test sample T was calculated using the following formula:

$$\eqalign{\rm Estimated \; activity \; of \; test \; Sample \; T \; (IU/mL) = { {{sample \; weight \; (mg) \times estimated \; potency \; (IU/mg)} \over {dilution \; volume \; (mL)}}}}$$

### Assay method and potency calculation

#### Anti-Xa factor potency assay

The anti-Xa factor potency assay was performed using a 96-well plate, with test solutions arranged as specified in the Supplemental Table. Each well was loaded with 16 μL of the test solution. To ensure accuracy and repeatability, all reagents were added using a multichannel pipette at a consistent speed and frequency, with thorough mixing after each addition. Initially, 16 μL of antithrombin III solution was added to each well, and the plate was incubated at 37 °C for 1 min to allow enoxaparin sodium to bind with antithrombin III and form a complex. Subsequently, 32 μL of factor Xa solution was added to each well, followed by a second incubation at 37 °C for 1 min to facilitate the reaction between factor Xa and the antithrombin III complex. Then, 80 μL of chromogenic substrate solution (S-2765) was added, and the plate was incubated at 37 °C for 4 min. During this step, unbound factor Xa catalyzed the hydrolysis of the substrate, producing a chromophore. The reaction was terminated by adding 120 μL of 30% acetic acid to each well, stabilizing the chromogenic product. Finally, the absorbance at 405 nm was measured using a Thermo Varioskan Flash spectrophotometer. The change in absorbance was inversely proportional to the anti-Xa factor potency.

The anti-Xa factor potency of enoxaparin sodium was calculated by measuring absorbance values and utilizing the linear relationship between the logarithm of absorbance and the concentration of the standard solutions. Specifically, linear regression analysis was performed, with the logarithm of absorbance as the dependent variable and the concentration of the standard or test solutions as the independent variable, to establish the regression model. Potency calculations were conducted using the 4 × 4 parallel line assay method (VØlund, 1978) for quantitative analysis. The BS 2000 software, developed by Shen Lianzhong and Li Bo from the National Institutes for Food and Drug Control (China), was used for potency calculations in this study. The results were expressed in International Units per milliliter (IU/mL). During the calculations, a correction for the sample’s moisture loss (3.9%) was applied to ensure the accuracy and consistency of the results. Two-tailed paired t-tests were performed using GraphPad Prism (version 9.2.0), with normality of paired differences confirmed by Shapiro-Wilk testing (*P* > 0.05 for all comparisons).

#### European Pharmacopoeia potency testing as control tests

The experiment (Council of Europe, 2024c) was conducted using 2.0 mL round-bottom microcentrifuge tubes. Each tube was initially loaded with 50 μL of the test solution and 50 μL of antithrombin III solution, thoroughly mixed, and incubated at 37 °C for 1 min. Subsequently, 100 μL of factor Xa solution was added to each tube, followed by another incubation at 37 °C for 1 min. Next, 250 μL of chromogenic substrate solution was added, and the tubes were incubated at 37 °C for an additional 4 min. The reaction was terminated by adding 375 μL of 30% acetic acid solution, stabilizing the final chromogenic reaction product. Upon completion of the reaction, the mixture was transferred to semi-micro cuvettes, and the absorbance at 405 nm was measured using a Shimadzu UV-2600 UV-Vis spectrophotometer. Data processing and result presentation were conducted as described in the methodology outlined above.

## Results

Using the described assay method, pH 7.4 buffer was used as a blank control (Blank). Absorbance measurements were performed for four different concentrations of enoxaparin sodium solutions (T1, T2, T3, and T4) and four different concentrations of standard solutions (S1, S2, S3, and S4). The raw absorbance data for all validation methods are provided in the Supplemental Table.

### Specificity, linearity, and range

The results are summarized in Table 3, which includes absorbance data for the pH 7.4 buffer (blank control, B), SC_Max_, S1, S2, S3, S4, SC_Min_, and T4. A comparison of the absorbance values for the blank control and other solutions revealed that the absorbance of the blank control was significantly higher than that of the other solutions. This finding indicates that the method can effectively distinguish between the blank control and test samples, demonstrating good specificity.

**Table 3:** Results of specificity, linearity, and range for the anti-Xa factor potency assay of enoxaparin sodium.

Solution	Labeled activity (IU/mL)	Absorbance	Comparison	Correlation coefficient R
B	Matrix: pH 7.4 buffer	0.9457	/	/
SC_Max_	0.192	0.2429	/	y = −0.75730x−0.30533 R = 0.9987
S_1_	0.16	0.2868	/	
S_2_	0.12	0.3898	/	
S_3_	0.09	0.4969	/	
S_4_	0.0675	0.5860	B > S_4_	
SC_Min_	0.054	0.6469	B > SC_Min_	
T_4_	/	0.5732	B > T_4_	/

Further analysis involved fitting the logarithm of absorbance values to the corresponding concentrations using linear regression. A linear equation was derived, and the correlation coefficient (R) was calculated. The results showed an R value of 0.9987, indicating a high degree of linearity between absorbance and solution concentration within the range of 0.054–0.192 IU/mL. These findings demonstrate that the method exhibits excellent linear characteristics within the studied concentration range.

### Robustness

The robustness assessment aimed to evaluate the stability and reliability of the assay results under minor variations in testing conditions. This study specifically examined the effects of changes in incubation temperature, incubation time, and the standing time of enoxaparin sodium solutions on assay performance.

For the incubation temperature assessment, tests were conducted at 39 °C (P1) and 35 °C (P2), and the results were compared with those obtained at the standard temperature of 37 °C (P0). Incubation time variation involved extending or shortening each reaction step’s incubation time by 15 s at 37 °C (corresponding to P3 and P4, respectively). Additionally, the impact of standing time was evaluated by allowing the sample solution to stand at room temperature for 24 h before re-dilution and testing. The potency of the solutions was then compared to that of freshly prepared samples.

The results demonstrated that changes in incubation temperature to 39 °C or 35 °C and variations in incubation time by ±15 s had minimal impact on the test sample potency, with relative standard deviation (RSD) values not exceeding 0.9% (Table 4). The potency of samples that stood for 24 h at room temperature (P24hr) showed an RSD of less than 2.0% when compared to freshly prepared samples (P0hr) (Table 4). These findings indicate that the assay method maintains good robustness under minor variations in temperature, time, and standing conditions, meeting the requirements for stability and reliability in testing.

**Table 4:** Robustness results of the anti-Xa factor potency assay for enoxaparin sodium under varying experimental conditions.

ID	Measured activity (IU/mg)	Dry matter potency (IU/mg)	RSD compared to P0
P_0_ (Initial)	102.36	106.51	/
P_1_ (+2 °C)	101.08	105.18	0.9%
P_2_ (−2 °C)	102.42	106.58	0.04%
P_3_ (+15 s)	100.99	105.09	0.10%
P_4_ (−15 s)	101.40	105.52	0.07%
P0hr	100.04	104.01	RSD compared to P0hr
P24hr	100.36	106.51	1.6%

### Evaluation of repeatability, intermediate precision, and reproducibility

This study evaluated the repeatability, intermediate precision, and reproducibility of the assay method. In the repeatability experiments, six replicate measurements of test sample solutions were performed within a single analytical run, with relative standard deviation (RSD) values all below 2.0% (Table 5), demonstrating good repeatability under identical conditions. Intermediate precision was assessed by a second operator measuring the potency of six test sample solutions on a separate day. The results also showed RSD values below 2.0%. A comparison with the initial measurements revealed consistent RSD values under 2.0%. A T-test comparison of the two datasets yielded a *P*-value > 0.7, indicating no statistically significant differences between the two sets of results (Table 5). This further confirmed the method’s precision and operational stability.

**Table 5:** Results and analysis of repeatability and intermediate precision for the anti-Xa factor potency assay of enoxaparin sodium (activity units: IU/mg).

ID		Weighing value (mg)	Estimated activity	Measured activity	Dry substance activity	RSD	RSD and *P* value
Repeatability	Re1	51.02	97.72	97.566	101.53	1.9%	1.7% *P >* 0.7
	Re2	50.97	97.81	100.68	104.77		
	Re3	50.25	99.21	99.047	103.07		
	Re4	50.70	98.33	99.328	103.36		
	Re5	51.54	96.73	97.140	101.08		
	Re6	50.14	99.43	102.18	106.33		
Intermediate precision	Re1	50.11	99.49	101.83	105.96	1.7%	
	Re2	51.30	97.18	99.718	103.76		
	Re3	49.93	99.85	99.301	103.33		
	Re4	50.02	99.67	101.38	105.49		
	Re5	50.24	99.23	98.42	102.41		
	Re6	50.15	99.41	97.598	101.56		

Reproducibility was assessed by conducting anti-factor Xa potency tests on three batches of enoxaparin sodium in two independent laboratories. The results showed high consistency between the two laboratories (Table 6). These findings strongly support the reproducibility of the method under different laboratory conditions.

**Table 6:** Detection results of two independent laboratories on three batches of enoxaparin sodium test samples for anti-Xa factor potency assay.

Anti-factor Xa activity (IU/mg)	YN001	YN002	YN003
Laboratory 1	104	110	107
Laboratory 2	106	104	104

### Accuracy assessment

The accuracy of the assay method was evaluated by adding known concentrations of standard solutions to the test sample solutions and calculating the recovery rates. The experimental results showed recovery rates ranging from 98.0% to 102.0%, indicating excellent accuracy in practical measurements. Additionally, the experiment was repeated six times, and the relative standard deviation (RSD) of the recovery rates remained below 2.0%, further validating the stability and reliability of the results (Table 7). These findings demonstrate that the method accurately reflects the actual activity of the samples within the expected range.

**Table 7:** Accuracy evaluation of the anti-Xa factor potency assay for enoxaparin sodium test samples (activity units: IU/mL).

ID	Measured activity	Standard added value	Measured activity after addition	Recovery rate	RSD
SP1 (Initial)	102.36 IU/mg	/	105.94 IU/ml	/	1.2%
PB1 (Spiking 1)	/	110 IU/ml	107.67 IU/ml	99.45%	
PB2 (Spiking 2)	/	110 IU/ml	107.13 IU/ml	98.47%	
PB3 (Spiking 3)	/	110 IU/ml	108.01 IU/ml	100.07%	
PB4 (Spiking 4)	/	110 IU/ml	108.31 IU/ml	100.62%	
PB5 (Spiking 5)	/	110 IU/ml	108.97 IU/ml	101.82%	
PB6 (Spiking 6)	/	110 IU/ml	108.55 IU/ml	101.05%	

### Validation of reliability using the European Pharmacopoeia method

The method described in the European Pharmacopoeia was used as a reference to validate the reliability of the assay. The experiment was conducted collaboratively by two operators, who independently measured the potency of six test sample solutions. The results showed that the relative standard deviation (RSD) of the potency measurements was consistently below 2.0%, indicating excellent precision of the method.

When compared to the repeatability data of the six test sample solutions, the RSD values were also below 2.0%, further confirming the stability and consistency of the assay results. A T-test was performed to compare the two datasets, yielding a *P*-value > 0.9, which indicated that the differences between the two groups were not statistically significant (Table 8). These findings validate the appropriateness of using the European Pharmacopoeia method as a reference and further support the reliability and reproducibility of the assay method developed in this study.

**Table 8:** Results and analysis of repeatability and control tests using the European Pharmacopoeia method for the anti-Xa factor potency assay of enoxaparin sodium (activity units: IU/mg).

ID		Weighing value (mg)	Estimated activity	Measured activity	Dry substance activity	RSD	RSD and *P* value
Repeatability	Re1	51.02	97.72	97.566	101.53	1.9%	1.3% *P >* 0.9
	Re2	50.97	97.81	100.68	104.77		
	Re3	50.25	99.21	99.047	103.07		
	Re4	50.70	98.33	99.328	103.36		
	Re5	51.54	96.73	97.14	101.08		
	Re6	50.14	99.43	102.18	106.33		
Control tests	Re1	49.92	99.87	98.813	102.82	0.6%	
	Re2	49.74	100.23	99.319	103.35		
	Re3	49.59	100.54	99.2	103.23		
	Re4	49.82	100.07	98.809	102.82		
	Re5	49.86	100.59	100.55	104.63		
	Re6	49.44	100.84	99.578	103.62		

## Discussion and conclusion

This study successfully validated the accuracy and reliability of the established method for determining the anti-factor Xa potency of enoxaparin sodium. The experimental results demonstrated that the method complies with the requirements for specificity, linearity, range, robustness, precision, and accuracy, as specified by ICH guidelines and the Chinese Pharmacopoeia. In robustness testing, the method exhibited low relative standard deviation (RSD) values even under minor variations in experimental conditions, such as changes in temperature or incubation time, underscoring its excellent robustness. Further experiments revealed high consistency in results between different operators, and strong agreement was observed between the results obtained from two independent laboratories. These findings confirm the method’s high reproducibility, making it suitable for inter-laboratory comparisons and method transfer. Moreover, the precision and accuracy validation results further affirmed the reliability of the method, highlighting its significance for ensuring the quality control of enoxaparin sodium. This validated method provides a solid foundation for consistent and reliable potency determination, supporting the broader goal of maintaining high standards of product quality and safety.

To further ensure the reliability of the results obtained in this study, the European Pharmacopoeia (Council of Europe, 2024c) method was selected as the control method. This choice was necessitated by the absence of a specific method for enoxaparin sodium in the Chinese Pharmacopoeia, leaving no domestic reference method available. The United States Pharmacopoeia (United States Pharmacopeia Convention, 2024) provides a range for the required volumes of test solutions and reagents rather than specific values, such an approach offers limited practical guidance and operability, and fails to meet the fundamental purpose of selecting a reference method—ensuring reproducibility and standardization. In contrast, The European Pharmacopoeia provides explicit procedural details and experimental parameters, significantly enhancing methodological clarity and operational feasibility. Consequently, the European Pharmacopoeia method emerges as the optimal choice for a control protocol.

The results demonstrated good agreement between the newly developed method and the European Pharmacopoeia method, indicating that the new method can serve as a reliable alternative.

Compared to the European Pharmacopoeia method, the new method significantly reduces the volume of samples and reagents required—only one-third of the original amounts—thereby lowering experimental costs. Additionally, the total reaction volume is minimized, enabling the experiment to be efficiently conducted using a 96-well plate. With rational plate design and the use of a multichannel pipette, reagent addition and mixing steps can be performed with greater operational efficiency and consistency, while also reducing the complexity of the procedure. This setup allows a single operator to complete the experiment, eliminating the need for two-person collaboration or expensive equipment. In contrast to traditional methods requiring individual reagent addition to microcentrifuge tubes followed by solution transfer to semi-micro cuvettes for absorbance measurement, the new method significantly simplifies the workflow. It enhances the consistency of each reaction step and eliminates the need for coagulation analyzers, further improving the method’s convenience and applicability. These advancements underscore the method’s suitability for practical applications while maintaining reliability and efficiency.

The potency of biological activity is calculated based on experimental data by comparing the activity differences between the test samples and the reference standard. In compliance with GMP/ICH regulatory requirements (International Conference on Harmonisation of Technical Requirements for Registration of Pharmaceuticals for Human Use, 2000), it is recommended to use national reference standards or other traceable reference materials to ensure consistency in test results across different laboratories. In this study, EP reference standards consistent with the European Pharmacopoeia method were utilized to provide inter-laboratory consistency. While numerous commercial test kits and point-of-care rapid detection methods are available (Al-Sallami & Medlicott, 2015; Salukhov et al., 2024), their use in pharmaceutical quality control must adhere to ICH requirements. In particular, traceable reference standards should be employed for calibration to ensure the accuracy and reliability of these methods, as emphasized in ICH Q6B (International Conference on Harmonisation of Technical Requirements for Registration of Pharmaceuticals for Human Use, 1999). This approach ensures that the methods used for quality assessment align with regulatory expectations and maintain high standards of precision and consistency.

Although this study achieved supportive conclusions, certain limitations remain. First, the inter-laboratory comparisons were limited to only three production batches due to constraints in the manufacturer’s production schedule, which may affect the statistical power of the results. Additional batch data from ongoing production will be required to enhance the reliability of the conclusions. Second, the Xa factor solution, as a biologically active reagent in this concentration-absorbance inverse relationship assay, may exhibit potency variations due to batch-to-batch differences or improper storage and transportation conditions. To mitigate this, pH 7.4 buffer was employed as a blank control (which does not consume Xa factor and therefore yields the highest absorbance). By maintaining the blank control absorbance within 0.8–1.0, optimal Xa factor concentration was ensured, enabling test solutions at different concentrations to demonstrate both good parallelism and distinct absorbance gradients. Overall, this study successfully validated a highly efficient, reliable, and reproducible method for determining the anti-Xa factor potency of enoxaparin sodium, with strong inter-laboratory reproducibility. The method provides manufacturers and regulatory authorities with an efficient, cost-effective, and user-friendly assay that serves as an objective and accurate tool for quality control, ensuring drug safety throughout its lifecycle. The results demonstrate that the method meets the relevant requirements of ICH guidelines and the Chinese Pharmacopoeia while exhibiting excellent performance in specificity, linearity, precision, accuracy, and robustness.

Furthermore, the methods and findings of this study provide valuable reference points for establishing quality standards and validating detection methods for other similar drugs (Yang et al., 2024). Through the optimization and validation of the experimental approach, the proposed method demonstrates excellent applicability. It is not only suitable for the quality control of enoxaparin sodium but also offers technical support for the development and quality evaluation of other low molecular weight heparins. Overall, the establishment of this method makes a significant contribution to ensuring drug quality and safety. It also holds substantial potential for broader application in the field of biopharmaceutical testing.

## Supplemental Information

10.7717/peerj.19437/supp-1 Supplemental Information 1 The raw data and arrangement of the solutions.

## Additional Information and Declarations

### Data Availability

The following information was supplied regarding data availability:

The raw measurements are available in the Supplemental File.
